# Prevalence and factors associated with intestinal parasitosis in children from an urban slum in Brazil: a cross-sectional study

**DOI:** 10.1590/1984-0462/2025/43/2024132

**Published:** 2024-12-20

**Authors:** Paulo Henrique Faria Domingues, Amanda Teixeira de Araújo, Isabella Pontes Silva, Arthur Vieira Soares, Rita de Cássia Ferraresso Alves, Lorâne Allen Andrade de Assis Vidal, Paula Caetano Araújo

**Affiliations:** a Instituto Master de Ensino Presidente Antônio Carlos Centro Universitário (IMEPAC), Faculdade de Medicina, Araguari, MG, Brazil.; b Exército Brasileiro, Laboratório de Análises Clínicas do 2^o^ Batalhão Ferroviário, Araguari, MG, Brazil.; c Universidade Federal de Uberlândia, Faculdade de Odontologia, Uberlândia, MG, Brazil.

**Keywords:** Intestinal diseases, parasitic, Parasitology, Poverty areas, Basic sanitation, Cross-sectional studies, Brazil, Enteropatias parasitárias, Parasitologia, Áreas de pobreza, Saneamento básico, Estudos transversais, Brasil

## Abstract

**Objective:**

This study aimed to estimate the prevalence and investigate the factors associated with intestinal parasitic diseases in children from an urban slum in Brazil.

**Methods:**

A cross-sectional study was conducted in children living in SEWA community, an urban slum located in Araguari, Minas Gerais, Brazil. The prevalence of intestinal parasitosis was determined via stool parasitological examination by spontaneous sedimentation. Socioeconomic, demographic, and behavioral data were collected to identify associated factors. The statistical analysis used the Poisson regression model, with robust variance for identification of associations.

**Results:**

Fifty-two children were interviewed, 41 of whom underwent parasitological examination. The prevalence of intestinal parasitosis was 43.9% (95% confidence interval — 95%CI 34.6–51.4), and 23% of children presented polyparasitism. *Endolimax nana*, *Entamoeba coli*, *Giardia lamblia* and *Strongyloides stercoralis* were identified in the stool samples. The adjusted analysis indicated negative associations of parasitosis with annual parasitological examination, possession of private health insurance, a mother who was married or in a stable relationship, and access to water treatment. Positive associations were observed with male sex, habit of playing with dirt, water ingestion from the hose, unemployed parents, low parental education, and the presence of a septic tank at home.

**Conclusions:**

The high prevalence of intestinal parasitosis in the SEWA community is a public health problem. The identification of modifiable and preventable factors highlights the need for interventions to improve living conditions not only for children but also for the entire community.

## RESUMO

**Objetivo:**

O estudo teve como objetivo estimar a prevalência e investigar os fatores associados às parasitoses intestinais em crianças de uma favela urbana brasileira.

**Métodos:**

Realizou-se um estudo transversal com crianças residentes na comunidade SEWA, uma favela urbana localizada no município de Araguari, Minas Gerais, Brasil. A prevalência de parasitose intestinal foi obtida pela análise parasitológica de fezes, utilizando o método de sedimentação espontânea. Dados socioeconômicos, demográficos e comportamentais foram coletados para identificar fatores associados. A análise estatística envolveu o modelo de regressão de Poisson, com variância robusta para a identificação de associações.

**Resultados:**

Foram entrevistadas 52 crianças, das quais 41 realizaram o exame parasitológico. A prevalência de parasitose intestinal foi de 43,9% (intervalo de confiança — IC95% 34,6–51,4), com 23% apresentando poliparasitismo. Parasitas como *Endolimax nana*, *Entamoeba coli*, *Giardia lamblia* e *Strongyloides stercoralis* foram identificados nas amostras. A análise ajustada indicou associações negativas com a realização anual do exame parasitológico, posse de plano de saúde privado, mãe casada ou em união estável e acesso ao tratamento de água. Associações positivas foram observadas com gênero masculino, hábito de brincar com terra, ingestão de água da mangueira, pais desempregados, baixa escolaridade dos pais e presença de fossa séptica em casa.

**Conclusões:**

A elevada prevalência de parasitose intestinal na comunidade SEWA configura-se como um problema de saúde pública. A identificação de fatores modificáveis e evitáveis ressalta a necessidade de intervenções para melhorar as condições de vida não apenas das crianças, mas de toda a comunidade.

## INTRODUCTION

Intestinal parasitic diseases, also known as intestinal parasitosis, are diseases triggered by helminths and protozoa and are emerging as a pressing challenge to public health. These pathologies not only affect individuals’ performance at school and at work but also put significant financial burdens on families through health care expenses.^1^ Areas of social and sanitary vulnerability are foci for the onset of morbidities, especially intestinal parasitic diseases, which are diseases closely associated with poverty and lack of basic sanitation.^2^ In Brazil, intestinal parasites are present in all regions, occurring mainly in rural areas and on the outskirts of urban centers, where sanitary infrastructure is deficient and the modes of transmission and prevention of these diseases are not well understood.^3^

In the context of intestinal parasitic diseases, schoolchildren are the ideal target for prevention and are the most important risk group.^4^ Children have frequent contact with contaminated soil and often have insufficient hygienic practices, becoming more susceptible. The adverse impact resulting from the absence of treatment in these children may not only retard their physical and mental development but also cause a wide range of symptomatic manifestations, such as diarrhea, abdominal pain, loss of appetite, weight loss, and even obstructive processes, leading, in some cases, to death.^4^

In Brazil, parasitic diseases are not subject to compulsory notification (with the exception of schistosomiasis),^5^ which limits the identification of their prevalence in the population. Underreporting and restricted routine screening directly impact the planning of public health policies.

The present study aimed to estimate the prevalence of and determine the factors associated with intestinal parasitic diseases in children from the SEWA community, an urban slum located in Araguari, Minas Gerais, Brazil.

## METHOD

A cross-sectional census-based study was conducted in children (0 to 12 years old) from the SEWA, an urban slum in the municipality of Araguari. Due to the lack of reliable cadastral information, the inhabitants of the area were censused, and the children of the community making up the target population investigated. The SEWA community is comprised of 85 individuals, including men, women, children and elderly people. The community consists of 32 families that do not have access to basic sanitation. Urban slums are defined as the irregular occupation of land owned by others that lacks these essential public services: garbage collection, sewage networks, water networks, electricity, and public lighting.^6^

The survey was conducted from June to October 2022. All children aged zero to 12 years in the community were invited to participate in the study. Previously trained interviewers approached the mother or, in her absence, the child’s legal guardian at their home. The participants responded to a questionnaire that was tested in a pilot study in ten individuals. The questionnaire mostly contained closed-ended questions and was organized into questions related to the children and their guardians, divided into distinct thematic sets.

The sample was obtained through a census; thus, epidemiological information was expected to be obtained for all children living in the SEWA community. To ensure we got a representative sample of the children in this community, the sample size was calculated using a confidence interval of 95% and a margin of error of 2.5%, estimating a prevalence of parasitic infections in children of 60%.^7^ Considering the population of 52 children in this community, a sample of 46 children was needed.

Data were collected on the social, demographic, and economic situation of the mother or, in her absence, of the child’s legal guardian, as well as information on the number of family members, type of housing, source of water for consumption, destination of sewage and trash, sanitary facilities, eating habits, prior knowledge about intestinal parasitic diseases, presence of self-reported diseases, and use of medication in the last seven days. Most of the questions in the questionnaire referred to the time of the interview to minimize recall bias. Additionally, 10% of the interviews were conducted by telephone to verify their authenticity.

Fecal samples were collected in wide-mouth plastic containers equipped with screw tops and collection paddles, while the respective collection bottles were properly labeled with the name of the child and their legal guardian and distributed to all the mothers of the selected children. The participants received detailed verbal instructions about the sample collection procedure. The research team made visits on two consecutive days, when necessary, to collect the samples. If the child was absent when we returned, a new collection was scheduled. This procedure was repeated once more in cases in which, on the second attempt, the recipients were again gone. During the collection period, an active search was also performed in the residences due to the long times it sometimes took to find the residents.

The samples were sent to the Clinical Analysis Laboratory of the 2^o^ Batalhão Ferroviário, a Brazilian Army unit in Araguari, Minas Gerais, to test for the presence of protozoan cysts, helminth eggs, and larvae via stool parasitological examination (SPE) using the spontaneous sedimentation method.^8^ Three slides were prepared from each fecal sample, and parasitological identification was conducted by light microscopy at 100× and 400× magnifications for visualization and confirmation of parasitic forms. The results of the children’s exams were sent to their mothers or legal guardians, and those with positive samples were referred to the basic health unit for appropriate treatment.

The data were tabulated in Microsoft Excel^®^ 2010 software and analyzed with Statistical Package for the Social Sciences (SPSS) software (version 21.0). Descriptive statistics of the variables are presented for the important aspects of the data to facilitate the understanding of the results. The prevalence of intestinal parasitosis among children in the community was estimated, and the proportions and their 95% confidence intervals (CIs) were calculated. Significant differences in descriptive variables between various groups of children were assessed using Pearson’s chi-squared test. To evaluate the association between intestinal parasitosis and the independent variables, a crude and an adjusted analysis were performed to obtain the prevalence ratios (PR) and their 95%CI. A Poisson regression model with robust variance was developed to identify factors associated with intestinal parasitic diseases. For all analyses, a significance level of 5% was set.

The project was approved by the Ethics Committee in Research with Human Beings of the Centro Universitário IMEPAC Araguari — Report No. 5339597 and Certificate of Presentation for Ethical Assessment (CAAE) No. 54493421.5.0000.8041 on August 4^th^, 2022. The data were collected only after the Minor’s Consent Form and Free and Informed Consent Form signed by the child’s legal guardian, as recommended by Resolution No. 466 of the Brazilian National Health Council (CNS) dated December 12, 2012.

## RESULTS

**Figure 1:**
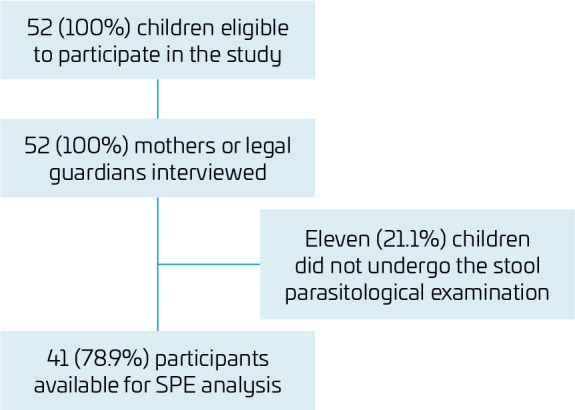
Process of recruitment, selection, inclusion, and evaluation of research participants.

Of the eligible children living in the SEWA urban slum, all 52 mother-child pairs (100%) accepted the invitation to participate in the study. Of the respondents, 41 (78.9%) underwent the SPE, and the prevalence of intestinal parasitosis was assessed (Figure 1). Twenty-six (50%) children were male, 86.5% were enrolled in day care or school, and the mean number of residents per household was 4.8 people (standard deviation — SD 1.7). The mean age was 6.1 years (SD 3.4), with a predominance of the age group >7 years (44.2%). Some 76.9% of the children had never undergone SPE, and only nine children (17.3%) had had verminosis once in their lifetime. Regarding the use of health services, a small portion of the sample (9.6%) had private health insurance, 48.1% had seen a health care provider in the last three months, and 11.5% had been hospitalized in the last 12 months.

SPE: stool parasitological examination.

All of the guardians who participated in the study were female. They were aged 18 to 65, with a mean age of 30.8 years (SD 8.4). The majority were single, widowed, or divorced (69.2%). The most common educational level was complete high school (42.3%), followed by complete elementary school or incomplete high school (30.8%), and only 2% of mothers had higher education. Almost all (96.1%) were in the ‘D-E’ social class, 57.7% reported not having occupational activity, and 78.8% received some type of government aid. Regarding housing, 82.7% lived in masonry homes with an internal bathroom for defecation (94.2%), but 96.1% did not have treated water, and the source of the water used was a clandestine connection. Regarding the origin of the water used for family consumption, only 15.4% of the participants performed water treatment. Only 78.8% of the families had a sewage system. Regarding pets, most families kept animals in their homes, and 56.1% of these had two to three pets (Table 1).

**Table 1:** Sociodemographic aspects of the sample studied in the population of the SEWA community, Araguari (MG), Brazil, 2022.

Characteristics	Children who underwent SPE (n=41)	Children who did not undergo SPE (n=11)	p-value
Children (0 to 12 years old)			
Male	20	6	0.910
Female	21	5	
Age group: 0–2 years	8	1	0.121
Age group: 3–6 years	14	6	
Age group: >7 years	19	4	
Enrolled in day care/school	35	10	0.089
At least one SPE per year	11	1	0.010
Intestinal parasitosis	5	4	0.561
Access to private health insurance	5	0	0.310
Medical appointment in the last 3 months	23	2	0.091
Hospitalization in the last year	5	1	0.421
Mothers or legal guardians			
Female	41	11	1.000
Age group: 18–30 years	20	5	0.812
Age group: 31–40 years	15	5	
Age group: 41–65 years	6	1	
Marital status: Married/stable union	12	4	0.633
Marital status: Single/widowed/divorced	29	7	
Education			
Illiterate and incomplete elementary school	11	2	0.040
Elementary school and incomplete high school	15	1	
Completed high school	14	8	
Higher Education and Graduate Studies	1	0	
Number of residents in the household: 2	3	0	0.271
Number of residents in the household: 3 a 4	20	5	
Number of residents in the household: 5 or more	18	6	
Economy class*			
C	1	1	0.940
D-E	40	10	
Unemployed^†^	22	8	0.133
Employed^‡^	19	3	
Government aid	32	9	0.563
Type of housing: Masonry	32	11	0.003
Type of housing: Canvas or tent	9	0	
Water treatment in the household	1	1	0.941
Origin of water: General distribution network	1	1	0.941
Origin of water: Clandestine connection	40	10	
Drinking water treatment	7	1	0.192
Treatment of sanitary sewage: Sewage system	40	1	<0.001
Treatment of sanitary sewage: Septic tank	1	10	
Place of defecation: Bathroom indoors	38	11	0.051
Place of defecation: Bathroom outdoors	3	0	
Presence of animals at home	35	6	0.161
Number of animals in residence: 1	9	2	0.059
Number of animals in residence: 2–3	21	2	
Number of animals in residence: 4 or more	5	2	

SPE: stool parasitological examination.

*Brazilian Economic Classification Criteria;.

^†^The variable ‘Unemployed’ includes unemployed individuals, retirees and students;

^‡^The variable ‘Employed’ includes housework, freelancer, informal work, government employee and private sector.

Table 1 compares the sociodemographic and environmental aspects of children who did and did not undergo parasitological examination and who were living in the community. There were no significant differences in economic aspects between the two groups. However, there are significant differences in the context of environmental aspects with regard to the type of housing they live in and the sanitary sewage treatment system adopted in their homes.

The prevalence of intestinal parasitosis was 43.9% (95%CI 34.6–51.4) in children from the SEWA subnormal cluster. The occurrence of polyparasitism (considering the presence of two or more pathogenic species or not) was 23%. There was a predominance of protozoa (90%) compared to helminths (10%), and four types of parasites were found in the children’s stool samples: *Endolimax nana* (n=14, 34.1%), *Entamoeba coli* (n=8, 19.5%), *Giardia lamblia* (n=5, 12.2%) and *Strongyloides stercoralis* (n=3, 7.3%). The number of samples positive for at least one pathogenic species was 19.5%.

The prevalence of intestinal parasitosis according to variables evaluated in children from the SEWA community is presented in Table 2 and 3. The analysis between intestinal parasitosis and the variables that were entered into the model was performed with the subjects who underwent the SPE. The adjusted model showed a negative association between the incidence of intestinal parasitosis and having taken at least one SPE per year (PR 0.48; 95%CI 0.28–0.61), private health insurance (PR 0.32; 95%CI 0.19–0.93), a mother who was married or in a stable relationship (PR 0.38; 95%CI 0.22–0.97), and access to water treatment at home (PR 0.77; 95%CI 0.18–0.91). A higher prevalence of helminths and protozoa was associated with male sex (PR 1.31; 95%CI 1.22–2.81), a habit of playing with dirt (PR 2.30; 95%CI 1.99–3.20), drinking water directly from the hose (PR 1.97; 95%CI 1.22–2.20), unemployed parents (PR 2.25; 95%CI 1.43–3.66), illiteracy or incomplete primary education (PR 2.08; 95%CI 1.06–2.29), and a septic tank for sewage treatment at home (PR 4.10; 95%CI 1.33–6.40).

**Table 2:** Prevalence of intestinal parasitic infections and prevalence ratios of associated factors in children from the SEWA community in the municipality of Araguari (MG), Brazil, 2022 (n=41).

Variable1	Prevalence of intestinal parasitosis % (95%CI)	Crude analysis		Adjusted analysis		p-value
		PR	95%CI	PR	95%CI	
Children of the SEWA community	43.9 (34.6–51.4)					
Factors related to children						
Sex						
Male	24.4 (20.7–27.6)	1.25	1.04–2.06	1.31	1.22–2.81	<0.01
Female	19.5 (13.9–23.8)	1.00	-	1.00	-	-
Age group (years)						
0–2	7.3 (6.4–9.2)	1.00	-	1.00	-	-
3–7	22.0 (19.5–25.3)	1.63	0.46–1.96	1.81	0.58–2.12	0.20
>7	14.6 (10.7–16.9)	0.90	0.39–1.67	0.65	0.25–1.47	0.28
At least one SPE per year*	7.3 (6.4–9.2)	0.20	0.15–0.44	0.48	0.28–0.61	<0.01
Presence of intestinal parasitosis in the last year*	2.4 (1.8–5.0)	0.06	0.02–0.80	0.19	0.08–1.13	0.79
Access to private health insurance*	2.4 (1.8–5.0)	0.06	0.02–0.30	0.32	0.19–0.93	<0.01
Medical appointment in the last 3 months*	21.9 (15.1–26.1)	0.99	0.59–1.33	1.03	0.42–1.24	0.34
Hospitalization in the last year*	2.4 (1.8–5.0)	0.06	0.03–1.05	0.33	0.12–1.09	0.97
Habit of nail biting*	14.6 (11.6–18.0)	0.49	0.38–1.21	0.53	0.40–1.23	0.59
Habit of washing fruits before eating*	22.0 (19.5–25.3)	1.01	0.85–1.39	1.11	0.90–1.55	0.79
Habit of playing with soil*	43.9 (34.6–51.4)	2.10	1.91–3.16	2.30	1.99–3.20	<0.01
Habit of washing hands after leaving the bathroom*	24.4 (20.7–27.6)	1.25	0.86–1.97	2.33	0.70–3.51	0.11
Habit of walking barefoot*	34.1 (29.8–38.0)	3.48	0.50–4.99	2.33	0.46–2.67	0.09
Habit of drinking water straight from the hose*	43.9 (34.6–51.4)	3.10	1.11–3.16	1.97	1.22–2.20	<0.01

SPE: stool parasitological examination; 95%CI: 95% confidence interval; PR: prevalence ratio.

*The comparison is made between the presence and absence of reference. For instance, the presence of intestinal parasitosis in the last year was compared to not having intestinal parasitosis.

**Table 3:** Prevalence of intestinal parasitic infections and prevalence ratios of associated factors in mothers or guardians from the SEWA community in the municipality of Araguari (MG), Brazil, 2022 (n=41).

Variable	Prevalence of intestinal parasitosis % (95%CI)	Crude analysis		Adjusted analysis		p-value
		PR	95%CI	PR	95%CI	
Children of the SEWA community	43.9 (34.6–51.4)					
Factors related to mothers or guardians						
Age group (years)						
18–30	26.9 (21.1–29.2)	2.10	0.89–4.89	1.24	0.53–2.84	0.51
31–40	14.6 (11.5–18.9)	1.90	0.78–4.62	1.21	0.52–2.54	0.56
41–65	2.4 (2.0–3.3)	1.00	-	1.00	-	-
Marital status						
Married/stable union	9.8 (4.8–13.4)	0.29	0.10–0.87	0.38	0.22–0.97	<0.05
Single/widowed/divorced	34.1 (29.8–38.0)	1.00	-	1.00	-	-
Education						
Illiterate and incomplete elementary school	7.3 (6.4–9.2)	1.54	0.87–2.72	2.08	1.06–2.29	<0.01
Elementary school and incomplete high school	17.1 (14.3–18.4)	1.14	0.56–1.94	1.36	0.73–2.18	0.56
Completed high school	19.5 (13.9–23.8)	1.04	0.88–2.87	1.13	0.92–1.87	0.16
Completed higher education	0.00	1.00	-	1.00	-	-
Number of residents in the household						
2	2.4 (2.0–3.3)	1.00	-	1.00	-	-
3–4	19.5 (13.9–23.8)	1.17	0.76–1.37	1.56	0.93–2.15	0.77
5 or more	21.9 (15.1–26.1)	1.23	0.42–1.99	1.93	0.51–1.99	0.56
Economic class						
C	2.4 (1.8–5.0)	1.72	0.44–1.83	1.21	0.55–1.39	0.33
DE	41.5 (32.8–46.4)	2.15	0.81–2.88	2.01	0.92–3.03	0.88
Work situation						
Unemployed	34.1 (29.8–38.0)	3.47	2.01–4.63	2.25	1.43–3.66	<0.01
Employee	9.8 (4.8–13.4)	1.00	-	1.00	-	-
Government aid*	39.0 (32.6–44.6)	7.96	5.21–10.3	2.11	0.87–3.91	0.88
Type of housing						
Masonry	39.0 (32.6–44.6)	1.00	-	1.00	-	-
Canva or tent	4.9 (2.0–6.8)	0.13	0.11–0.80	0.88	0.22–1.31	0.15
Water treatment*	0.00	0.04	0.02–0.59	0.77	0.18–0.91	<0.05
Origin of water used in the household						
Public water supply system	0.00	0.04	0.02–0.59	0.91	0.39–1.03	0.07
Clandestine connection	43.9 (34.6–51.4)	1.00	-	1.00	-	-
Drinking water treatment*	2.4 (1.7–5.9)	0.05	0.02–0.81	0.24	0.12–2.81	0.89
Presence of septic tank in the household*	43.9 (34.6–51.4)	22.4	18.6–31.1	4.10	1.33–6.40	<0.01
Presence of animals in the household*	36.6 (29.9–40.6)	5.0	0.88–6.10	4.46	0.99–5.13	0.06
Number of animals in residence						
1	7.3 (5.8–8.2)	1.00	-	1.00	-	-
2–3	24.4 (21.3–27.1)	1.54	0.87–2.72	1.38	0.77–2.29	0.42
4 or more	4.9 (2.8–5.3)	1.04	0.56–1.99	1.22	0.72–2.11	0.38

95%CI: 95% confidence interval; PR: prevalence ratio.

*The comparison is made between the presence and absence of reference. For instance, the presence of intestinal parasitosis in the last year was compared to not having intestinal parasitosis.

## DISCUSSION

The results of this study revealed that intestinal parasites are present in 43.9% of children living in the SEWA community. Among these children, 23% had polyparasitism, and 19.5% tested positive for at least one pathogenic species. The adjusted regression model showed a negative association between the incidence of intestinal parasitosis and at least one SPE per year, private health insurance, a mother who was married or in a stable union, and access to water treatment at home. In turn, intestinal parasitosis was more frequent among male children, children who had the habit of playing with dirt, drinking water directly from the hose, and parents who were unemployed, illiterate, or had not completed elementary school.

A systematic review of intestinal parasitic infections in Brazil found a similar prevalence (46%) of intestinal parasitic infections as reported earlier among children from the SEWA community.^9^ The prevalence of intestinal parasitosis found in the present study was also higher than the prevalence reported in some studies conducted in the state of Minas Gerais^10-14^ and in the southeastern region of Brazil,^9^ studies that exhibit certain methodological similarities with the present one. A possible explanation for these discrepancies in values could be the close relationship between unfavorable socioeconomic conditions and the prevalence of intestinal parasites in subnormal communities.^15,16^ Compared to other studies conducted in the Triângulo Mineiro Mesoregion, the present study also showed a higher prevalence of intestinal parasitosis than other studies conducted with inhabitants of Araguari (38%)^17^ and institutionalized children in the region of Uberlândia (29%).^13^ Compared to children living in an urban slum in Uberlândia, the prevalence rate (45%) was close to the result (44%) among children from the SEWA community in Araguari.^16^ The high prevalence of intestinal parasitic diseases in these environments, especially among children, is in agreement with studies that show the direct influence of the socioenvironmental context on the spread of these diseases.^18^

The parasite species we identified are significant from both an epidemiological and a clinical point of view due to their impacts on health in general. These parasites are considered the primary elements that cause debilitation and are often linked to the occurrence of chronic diarrhea and malnutrition, negatively affecting physical and cognitive development, especially in children.^19^ An integrative review examined the factors associated with intestinal parasitic infections in elderly individuals in Brazil and found that the most common protozoa in the included studies were *Entamoeba coli, Endolimax nana*, and *Giardia lamblia*.^20^ These were also the three most common protozoa in our study, suggesting that although *E. coli* and *E. nana* are normally present in the human intestine without causing damage, their detection may indicate exposure to fecal contamination, suggesting a possible lack of basic hygiene in certain populations.^21^ In some studies of children, the parasite most frequently found was *G. lamblia*.^16,22,23^ The present study found *G. lamblia* in a significant number of patients, although this number was considerably lower than others have found. The results regarding *G. lamblia* infection could have been different if we had used the FAUST method, which is specific for the diagnosis of protozoa. On the other hand, the presence of *G. lamblia* may be related to the proximity of the fungus to infected domestic animals due to the zoonotic capacity of this protozoan.^24^ The proportion infected by *Strongyloides stercoralis* (7.3%) reinforces the high incidence of this parasite in the Triângulo Mineiro area,^16,25^ which can be explained by the children’s habit of playing with dirt, which lets the larvae of this parasite penetrate the skin. This result is relevant because rates above 5% make a parasite endemic.^26^

A total of 23% of the children had polyparasitism, higher than the 7.3–15.7% found in other studies.^16,17^ This situation might be favored by the sanitation and hygiene conditions found in the SEWA community.

The presence of intestinal parasitosis was negatively associated with having at least one SPE per year and with access to private health insurance, possibly explained by the children’s greater use of health services. This relationship has been observed in Brazilian studies in a similar context.^15,27^ This highlights the influence of regular medical care in the prevention and control of parasitic diseases and the importance of early detection and timely treatment to interrupt transmission and reduce the burden of these diseases in the community.

The present study revealed that children who play with dirt, drink water directly from the hose, have unemployed, illiterate, or sub-elementary-educated parents, or live at a home with a septic tank are more susceptible to intestinal parasitic diseases. These risk factors, such as hygiene habits during play, housing conditions, and educational and occupational levels, are in line with the findings of previous studies that indicate the importance of these aspects in the spread of intestinal parasitic diseases.^28^ The association between the consumption of untreated water and the presence of intestinal parasites reinforces the importance of basic sanitation in the prevention of these diseases. The absence of adequate water treatment and dependence on untreated sources considerably increase the risk of parasitic infections.^29^ In the present study, a higher prevalence of intestinal parasitosis was observed in male children, disagreeing with the results found in the literature.^16^

Some limitations are inherent to the design of the present study and deserve attention. There may be information bias because some data on the health status and habits of the children were obtained through the self-reports of mothers without objective confirmation. Another limitation is that we ran the SPE on a single sample of each child. The feasibility of detecting parasites in the SPE is enhanced by the analysis of multiple samples due to factors such as the intermittent excretion of certain parasites, notably protozoa; the irregular distribution of helminth eggs in the feces; and the different stages of protozoa. Conventionally, the collection of three samples on consecutive or alternate days is recommended to minimize the influence of the parasite cycle.^30^ However, our study boasts a greater participation rate of the children than other studies have achieved in this population.^15^ The method of spontaneous sedimentation used in this study was not specifically intended for the diagnosis of certain species of intestinal parasites. We chose it because of its effectiveness as an appropriate tool for triage, in addition to being commonly used in public health networks. Because of this choice, we may have underestimated the prevalence of intestinal parasitic diseases in children from the SEWA community or failed to find infections due to the technical approach adopted.

The strengths of this study include the fact that this is the first cross-sectional census-based study of the SEWA community, which is highly relevant for mitigating the risk of infection by enteroparasites, especially given the lack of basic sanitation in the region. The results are made more valid by the methodological care applied, the standardized study procedures, the testing of the questionnaire in a pilot study, and the auditing of 10% of the interviews.

In areas with high prevalence of parasitic infections, as well as regions with inadequate sanitation, water supply, and sewage treatment, it is essential to develop strategies for controlling parasitic diseases. In addition to preventive measures such as access to basic sanitation and health education, regular stool examinations are recommended when there are clinical suspicions of intestinal parasitic infections.^3,31^ Early diagnosis and specific treatment of causative agents are crucial to reduce the spread and impact of infections. Periodic collection of multiple stool samples can improve the detection of parasites with intermittent excretion.^3,31^

Periodic campaigns of chemoprophylaxis are an effective preventive measure for reducing parasitic load and its consequences, as recommended by the World Health Organization and the Brazilian Ministry of Health.^3,31^ Preventive treatment of enteroparasitosis in school-aged children between five and 14 years old, through the administration of broad-spectrum medications such as albendazole, can reduce both the prevalence and intensity of infection. Depending on the community’s epidemiological situation, chemoprophylaxis should be applied more or less frequently and to other vulnerable populations.^3^ Additionally, the implementation of continuous educational programs in schools and communities, focusing on the importance of personal hygiene, sanitation practices, and prevention methods, is essential for the prevention, control, and reduction of the burden of parasitic infections.

Intestinal parasitosis were a high prevalence among children from the SEWA community in Araguari, Minas Gerais. The results highlight the complexity and interdependence of socioeconomic, environmental, and behavioral factors in the occurrence and spread of intestinal parasitic diseases. The factors that contribute to the parasitic infections are preventable and subject to modification. The implementation of adequate conditions of basic sanitation and the availability of drugs that are easy to administer for the treatment of intestinal parasitosis are fundamental elements for all communities that seek to reduce the prevalence of parasitism. The results may suggest specific targets for the development of treatment strategies, access to health services, and education, with the objective of reducing the incidence of parasitic diseases in vulnerable communities.

## Data Availability

The database that originated the article is available with the corresponding author. CAAE: 54493421.5.0000.8041
